# The value of lymph nodes ratios in the prognosis of resectable remnant gastric cancer through the retrospective propensity score matching analysis

**DOI:** 10.1186/s12957-023-03137-z

**Published:** 2023-08-11

**Authors:** Biao Yang, Tao Liu, Hangtian Cui, Zhengmao Lu, Guoen Fang, Xuchao Xue, Tianhang Luo

**Affiliations:** 1 Department of General Surgery, Changhai Hospital, Second Military Medical University/Naval Medical University, Shanghai, 200433 China; 2 Department of Emergency, Changhai Hospital, Second Military Medical University/Naval Medical University, Shanghai, 200433 China

**Keywords:** Remnant gastric cancer, Clinicopathological characteristics, Overall survival, Lymph nodes ratio

## Abstract

**Purpose:**

Currently, the characteristics and prognosis of remnant gastric cancer (RGC) are not fully understood yet. The present study aimed to describe the details of clinicopathological features of resectable RGC and investigated the factors affecting survival after the curative operation.

**Methods:**

From Jan. 2006 to Dec. 2015, a total of 118 resectable RGC patients (the RGC group) and 236 age-, sex- and TNM stages-matched resectable gastric cancer (GC) patients (the control group) were recruited retrospectively. Clinicopathological characteristics and overall survival were compared between the two groups.

**Results:**

The overall survival rate was 46.61% for RGC patients compared to 55.08% for control groups (*P* < 0.01), and the mean overall survival time of RGC patients was 40.23 ± 32.27 months, compared to 55.06 ± 34.29 months in the control group (*P* = 0.023 after matching). The overall survival (OS) of RGC patients with stage IIb was much worse than IIa (*P* < 0.001) and similar to IIIa (*P* = 0.463) and IIIb (*P* = 0.014). Multivariate Cox proportional hazards model analysis revealed that TNM stage (HR: 3.899, *P* < 0.001) and lymph nodes ratio (LNR) (HR: 2.405, *P* = 0.028) were independent prognostic significance to OS.

**Conclusions:**

The OS of RGC was much worse than GC with similar TNM stages, and LNR might consider a highly reliable indicator to evaluate the prognostic in RGC.

## Introduction

Residual gastric cancer (RGC) is a specific type of gastric cancer, that originally refers to cancers after gastric resection for either benign or malignant diseases, which develops 5 years following the initial procedure [1–3]. After continuous research and development, cancerous changes that occur in the stomach more than 10 years after partial gastrectomy due to malignant cancers have also been included in the scope of RGC [4]. RGC differs from the concept of "carcinoma in the remnant stomach (CRS)" proposed by the Japanese Gastric Cancer Association [5, 6]. Regarding the exact definition of RGC, there is currently no consensus worldwide. According to the 2018 Surgical Expert Consensus on the Definition of Residual Gastric Cancer in China, the RGC in clinical work is defined as new cancer that occurs in the residual stomach more than 5 years after gastrectomy for benign diseases or more than 10 years after gastrectomy for gastric cancer (GC) [7]. With the increasing number of patients undergoing gastrectomy, the incidence of RGC is increasing year by year [8]. Therefore, it is necessary to further strengthen the understanding of the clinical characteristics of RGC and improve the level of diagnosis and treatment.

At present, most scholars believe that the main pathogenesis of RGC is related to the anatomical changes of the residual stomach after partial gastrectomy, and the reflux stimulation of bile, intestinal juice, pancreatic juice, and other alkaline fluids [9]. This leads to the development of the residual gastric mucosa into chronic atrophic gastritis, intestinal metaplasia, and dysplasia, which may eventually lead to RGC [10]. The interval between RGCs after surgery for benign gastric disease is longer than for malignant disease. The time for RGC to develop after resection of benign gastric disease is 20–36 years, and the time after resection of malignant disease is 2–9 years [11–13]. The incidence of RGC is higher in European and American populations than in Asian populations. This may be due to the higher incidence of primary gastric diseases (including primary GC) in Asia than in other parts of the world [3, 14, 15]. With the continuous improvement of the early diagnosis rate of GC, the overall survival time of patients after surgery has been significantly prolonged, and the incidence of RGC after radical subtotal gastrectomy has increased year by year. Due to the atypical clinical manifestations of early RGC patients, most of them have progressed at the time of consultation. A study in Europe suggests that RGC needs to be addressed early and properly [16]. Therefore, the early diagnosis and treatment of RGC are particularly important.

Clinical manifestations in patients with RGC are generally non-specific and are easily missed or misdiagnosed by clinicians. Early RGCs generally have no obvious clinical signs and symptoms and often require gastroscopy to confirm the diagnosis. In clinical practice, RGC has a higher rate of invasion to adjacent organs, and lymph node metastasis was commonly observed [17], which may cause the prognosis of RGC much worse than primary GC [18]. But some articles argued that the prognosis of RGC was similar to primary GC [19]. Several previous studies have discussed the clinical characteristics of resectable RGC in a limited number of cases, but factors influencing the prognosis of patients with RGC still remained unclear or controversial [20–22]. In this study with a relatively larger sample size, we described the clinicopathological characteristics of resectable RGC and investigated factors affecting prognosis after the curative operation, attempting to address the inherent controversies more reliably.

## Methods

### Study design and participants

From January 2010 to December 2015, the clinicopathological data of 195 patients with RGC who underwent curative gastric resection (including total resection of the gastric stump with lymphadenectomy) at Changhai Hospital were retrospectively collected. In this study, RGC was defined as a carcinoma arising in the gastric remnant after gastrectomy, regardless of the histology of the previous lesion (benign or malignant), its risk of recurrence, the extent of initial resection, or methods of reconstruction [23]. The TNM classification (7th edition) was reported as a practical staging system for RGC, so the RGC stage in this study was conducted according to the TNM classification (7th edition) by AJCC [24, 25]. All the patients enrolled received standard therapy, which meant curative operation and postoperative adjuvant chemotherapy if necessary according to the NCCN Clinical Practice Guidelines in Oncology Gastric Cancer. This study was approved by the Medical Ethics Committee of Changhai Hospital of the Second Military Medical University. The informed consent requirement was exempted.

Inclusion criteria: 1) age 18 to 85 years, life expectancy > 3 months; 2) Histopathological examination confirmed as stomach adenocarcinoma; 3) Benign gastric lesions above 5 years after the initial partial gastrectomy, new cancers on the remnant gastric; 4) gastric malignant lesions undergo partial gastric resection and more than 5 years after R0 resection, new cancers of the remnant gastric; 5) complete clinical-pathological data; 6) adequate organ functions (leukocyte count > 3,500/μl, platelet count > 100,000/μl, hemoglobin > 10.0 g/dl, serum creatinine < 1.25 times the upper limit of normal (ULN), transaminases and alkaline phosphatases < 2.5 times ULN or < 5 times ULN in patients with liver metastasis, bilirubin < 1.5 times ULN, and prothrombin time < 12.0 s). Exclusion criteria: 1) concomitant other primary cancers; 2) incomplete or unavailable medical record data, incomplete preoperative examination, or refusal of surgical treatment; 3) patients whose initial lesions are malignant and have not been removed by R0; 4) patients with incomplete medical records.

Between January 2010 and December 2015, a total of 195 patients with RGC and 2001 patients with GC were admitted. Under case selection criteria, a total of 77 patients with RGC and 624 patients with GC were excluded. Reasons for ineligibility included patients incompatible with the operation (8/71 cases), positive margin (3/41 cases), inadequate organ function (4/155 cases), incomplete clinical data (6/162 cases), palliative operation or M1 stage (51/195 cases), and wedge gastrectomy (5/0 cases). Finally, this study included 118 resectable RGC patients and 1377 patients with GC who performed the curative operation.

### Follow-up and data collection

Follow-ups were conducted once every 28-day for half a year, once every 3-month for 2 years, once every 6-month for 3 years, and yearly thereafter. Follow-up terms consisted of physical examination, complete blood test, chest radiography, and ultrasound of the abdomen as clinically indicated. Computed tomography (CT) scanning or magnetic resonance imaging (MRI) would be performed if necessary. The follow-up data of patients were collected either through telephone or outpatient service.

Medical records of patients enrolled were collected retrospectively by two assistants from Medbanks Network Technology CO. Collected data included patients’ background, time intervals between primary and secondary operation, pathologic diagnosis of RGC, surgical data, and tumor characteristics. The data errors were corrected by the Department of General Surgery Changhai Hospital.

### Statistical analysis

To minimize the influence of other confounders on the outcome, we used a propensity score analysis to match RGC patients with GC patients. RGC patients were matched in a 1:4 ratio with GC patients using the nearest neighbor matching and based on gender (male or female), age (≤ 60 or > 60 years), tumor Grade (well-differentiated, moderately differentiated, poorly differentiated), tumor size (≤ 3 cm or > 3 cm), number of lymph nodes (LN) harvested, TNM stage (7th edition, AJCC) and pathological lymph nodes positive ratio (pLNR, number of positive LN/number of resected LN).

Statistical analysis was performed using SPSS software (18th version, SPSS, Chicago, IL, USA). Enumeration data were presented in percentage, measurement data was presented in average number and standard deviation, and abnormal distribution data was displayed by median and quartile. The associations between categorical variables were analyzed by the Pearson chi-square test. Continuous variables were assessed using t test or Cochran-Mantel–Haenszel test as appropriate. Survival curves and univariate analysis were performed in accordance with the Kaplan–Meier method, and the log-rank test was used to evaluate statistical significance. Cox regression analysis was used in multivariate analysis to assess independent prognostic factors. A probability (P) value < 0.05 was considered to indicate statistical significance. All tests were two-sided.

## Results

### Enrollment and patient characteristics

**Fig. 1:**
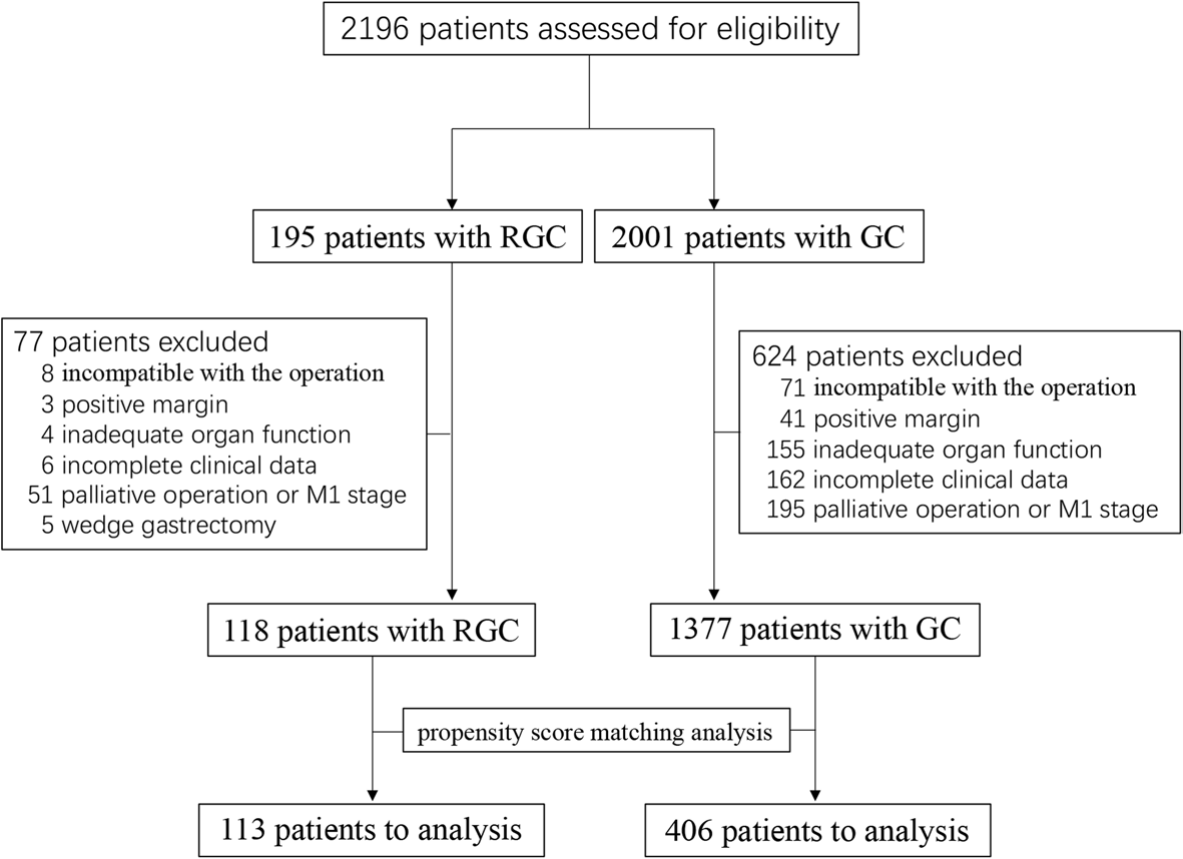
Flow diagram of the selection of study participants

The flowchart of the program for participants in this study is shown in Fig. 1. The baseline characteristics of patients with GC in the pre-match and post-match samples are presented in Table 1. The two groups did not differ significantly in terms of age, sex, tumor grade, and TNM stage. However, compared with the control group, the RGC group had significantly larger tumors, higher pLNR, and fewer LNs harvested (all *p* < 0.05).

**Table 1:** Clinicopathological features of RGC and GC before and after matching on the propensity score

Clinicopathological Features	Before matching		*P*-value	After matching		*P*-value
	**RGC (*n* = 118)**	**GC (*n* = 1377)**		**RGC (*n* = 113)**	**GC (*n* = 406)**	
**Gender**			0.0088^#^			0.864^#^
**female**	22 (18.64%)	414 (30.07%)		22 (19.47%)	82 (20.20%)	
**male**	96 (81.36%)	963 (69.93%)		91 (80.53%)	324 (79.80%)	
**Age (year)**			0.0258^#^			0.760^#^
** ≤ 60**	49 (41.53%)	719 (52.21%)		48 (42.48%)	179 (44.09%)	
** > 60**	69 (58.47%)	658 (47.79%)		65 (57.52%)	227 (55.91%)	
**Tumor Grade**			0.0031^#^			0.823^#^
**poorly**	70 (59.32%)	639 (47.47%)		66 (58.41%)	227 (55.91%)	
**moderately**	38 (32.20%)	642 (47.70%)		37 (32.74%)	135 (33.25%)	
**well**	10 (8.47%)	65 (4.83%)		10 (8.85%)	44 (10.84%)	
**Tumor size**			0.0372^#^			0.014^#^
** ≤ 3**	39 (33.05%)	591 (42.92%)		39 (34.51%)	193 (47.54%)	
** > 3**	79 (66.95%)	786 (57.08%)		74 (65.49%)	213 (52.46%)	
**Mean of LN Harvested**			< 0.0001*			< 0.0001*
	13.13 ± 9.58	19.57 ± 5.59		13.34 ± 9.67	19.35 ± 5.40	
**pT stage**			0.0103^†^			0.484^†^
**T1**	28 (23.73%)	212 (15.40%)		28 (24.78%)	93 (22.91%)	
**T2**	12 (10.17%)	412 (29.92%)		12 (10.62%)	49 (12.07%)	
**T3**	47 (39.83%)	644 (46.77%)		42 (37.17%)	156 (38.42%)	
**T4**	31 (26.27%)	109 (7.92%)		31 (27.43%)	108 (26.60%)	
**pN stage**			< 0.001^†^			0.294^†^
**N0**	51 (43.22%)	453 (32.90%)		51 (45.13%)	174 (42.86%)	
**N1**	27 (22.88%)	256 (18.59%)		25 (22.12)	79 (19.46%)	
**N2**	17 (14.41%)	285 (20.70%)		17 (15.04)	64 (15.76)	
**N3**	23 (19.49%)	383 (27.81%)		20 (17.70%)	89 (21.92%)	
**TNM stage**			0.5478^†^			0.161^†^
**Stage I**	39 (33.05%)	350 (25.42%)		39 (34.51%)	150 (36.95%)	
**Stage II**	22 (18.64%)	457 (33.19%)		20 (17.70%)	110 (27.09%)	
**Stage III**	57 (48.31%)	570 (41.39%)		54 (47.79%)	146 (35.96%)	
**pLNR**			< 0.0001^#^			< 0.0001^#^
**0**	51 (43.22%)	453 (32.90%)		51 (45.13%)	174 (42.86%)	
**0〜0.5**	32 (27.12%)	592 (42.99%)		28 (24.78%)	184 (45.32%)	
** ≥ 0.5**	35 (29.66%)	332 (24.11%)		34 (30.09%)	48 (11.82%)	

*RGC* remnant gastric cancer, *GC* gastric cancer, *LN* lymph nodes, *T* tumor, *N* node, *M* metastasis, *LNR* lymph nodes ratio

^#^chi-square test

^*^t-test

^†^Cochran-Mantel–Haenszel test

### Survival analysis of RGC

**Fig. 2:**
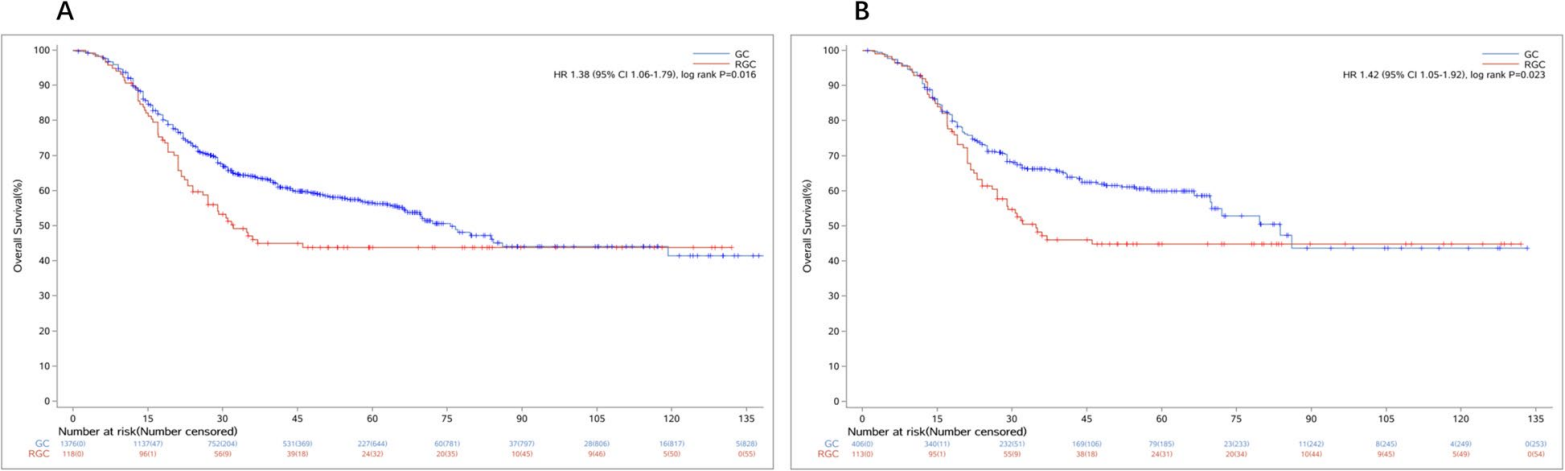
The OS curves of RGC and GC. **A** The OS curves of RGC and GC before matching. **B** Overall survival curves of RGC and GC after matching. RGC, remnant gastric cancer; GC, gastric cancer; OS, overall survival

**Fig. 3:**
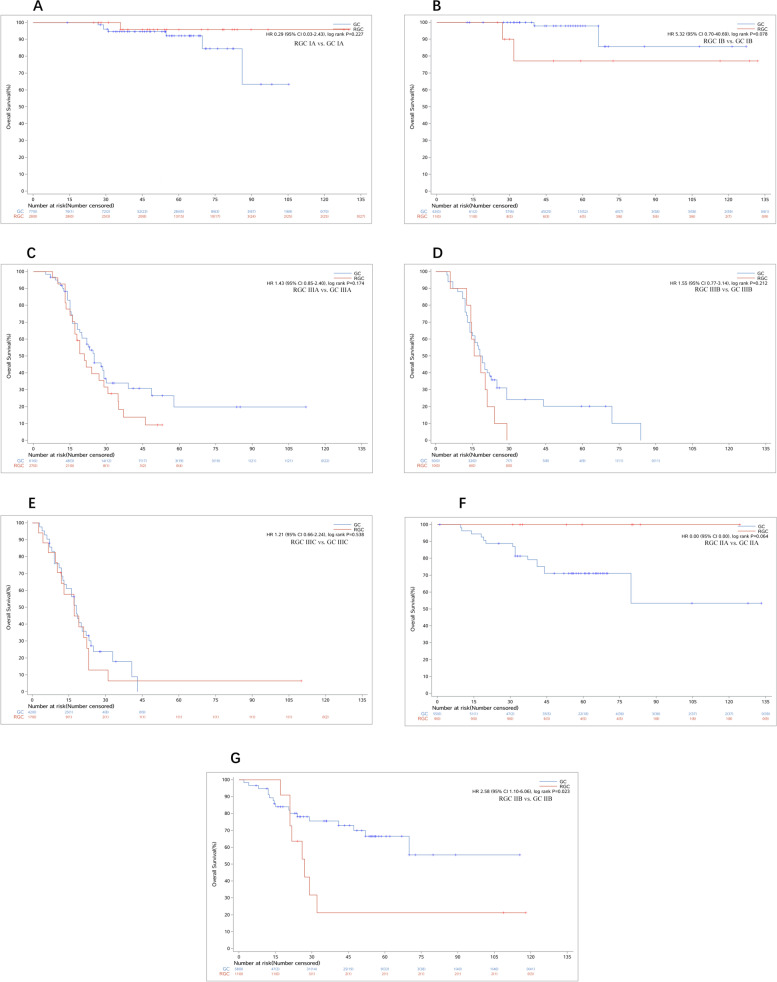
The OS curves of RGC and GC are stratified by the TNM stage. **A**, **B** The OS of RGC with stage I was similar to GC (*p* = 0.227; 0.078). **C-E** The OS of RGC with stage III was similar to GC (*p* = 0.174; 0.212; 0.538). **F**, **G** The OS of RGC with stage II was worse than GC (*p* = 0.064; 0.023). RGC, remnant gastric cancer; GC, gastric cancer; OS, overall survival; TNM, tumor node metastasis

The overall survival rate was 46.61% for RGC patients compared to 55.08% for control groups (*P* < 0.01), and the mean overall survival time of RGC patients was 40.23 ± 32.27 months, compared with 55.06 ± 34.29 months in the control group (*P* = 0.023 after matching) (Fig. 1). As shown in the OS curves, the OS rates of RGC patients with TNM stage I (92.31%) and stage III (14.81%) were almost similar to GC patients (91.33% and 20.51%) (Fig. 2A-E). But the OS rate of RGC patients with TNM stage II was 45.00%, which was much worse than the control group (75.45%) (Fig. 2F, G). Another interesting finding showed that there was no significant difference in OS rates among RGC patients with TNM stage IIb, GC patients with TNM stage IIIa (*P* = 0.736), and IIIb (*P* = 0.105) (Fig. 3).

### Association between primary disease and clinicopathological characteristics

To find the relationship between primary disease and clinicopathological characteristics in RGC, all RGC patients were divided into two sub-groups (RGC-B for primary benign disease vs. RGC-M for primary malignant disease). Primary benign disease meant benign ulcer, while primary malignant disease referred to gastric adenocarcinoma. In all 118 cases, 61 (51.69%) RGC patients had primary GC in the first operation. The time interval, number of LN harvested, and pN stages were statistically different between the two groups (Table 2). Theoretically speaking, the primary disease was an important personal factor so it may influence the prognosis, however, our study showed there were no obvious survival differences attributed to it (40.99 ± 33.50 months vs. 39.52 ± 31.34 months; *P* = 0.26).

**Table 2:** Association between primary disease and clinicopathological features

Clinical features	RGC-B (*n* = 57)	RGC-M (*n* = 61)	*P*-value
**Gender**			0.271^#^
**female**	9 (15.79%)	13 (21.31%)	
**male**	48 (84.21%)	48 (78.69%)	
**Age (year)**			0.062^#^
** ≤ 60**	21 (36.84%)	28 (45.90%)	
** > 60**	36 (63.16%)	33 (54.10%)	
**Time interval(month)**			< 0.001^*^
	151.16 ± 76.30	68.05 ± 32.05	
**Tumor location**			0.445^#^
**anastomosis**	29 (50.88%)	31 (50.82%)	
**non-anastomosis**	28 (49.12%)	30 (49.18%)	
**Tumor Grade**			0.122^†^
**poorly**	37 (64.91%)	33 (54.10%)	
**moderately**	17 (29.82%)	21 (34.43%)	
**well**	3 (5.26%)	7 (11.48%)	
**Tumor size**			0.251^#^
** ≤ 3**	35 (61.40%)	32 (52.46%)	
** > 3**	22 (38.60%)	29 (47.54%)	
**Mean of LN Harvested**			< 0.001^*^
	19.86 ± 7.46	6.84 ± 6.61	
pT* stage			0.189^†^
**T1**	13 (22.81%)	15 (24.59%)	
**T2**	7 (12.28%)	5 (8.20%)	
**T3**	24 (42.11%)	23 (37.70%)	
**T4**	13 (22.81%)	18 (29.51%)	
pN* stage			0.028^†^
**N0**	23 (40.35%)	28 (45.90%)	
**N1**	9 (15.79%)	18 (29.51%)	
**N2**	10 (17.54%)	7 (11.48%)	
**N3**	15 (26.32%)	8 (13.11%)	
**TNM stage**			0.387^†^
**Stage 1**	19 (33.33%)	20 (32.79%)	
**Stage 2**	10 (17.54%)	12 (19.67%)	
**Stage 3**	28 (49.12%)	29 (47.54%)	

*RGC-B* remnant gastric cancer primary benign disease, *RGC-M* primary malignant disease, *RGC* remnant gastric cancer, *GC* gastric cancer, *LN* lymph nodes, *T* tumor, *N* node, *M* metastasis, *LNR* lymph nodes ratio

^#^chi-square test

^*^t-test

^†^Wilcoxon rank sum test

### The effects of Lymph Nodes Ratio (LNR)

**Fig. 4:**
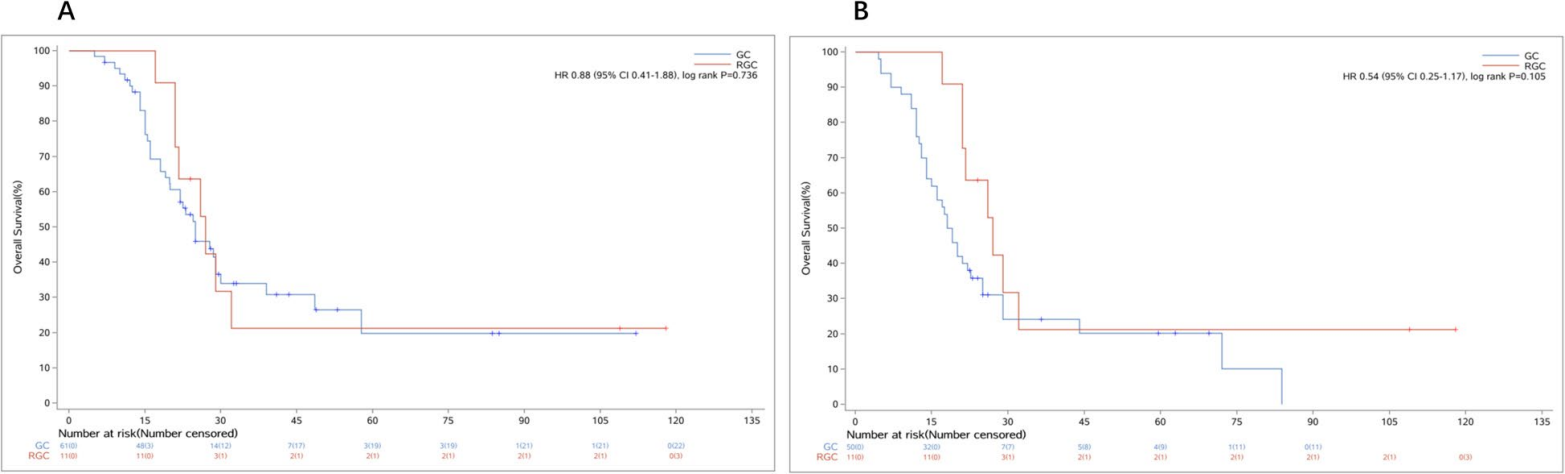
The OS curves of RGC with stage IIb. **A** The OS of RGC with stage IIb was similar to GC with stage IIIa (*p* = 0.736). **B** The OS of RGC with stage IIb was similar to GC with stage IIIb (*p* = 0.105). RGC, remnant gastric cancer; GC, gastric cancer; OS, overall survival

There was an interesting finding that the OS of RGC patients with TNM stages II varied much from each other (Fig. 2A, B). The OS of patients with stage IIb was much worse than IIa (*P* < 0.001) and similar to IIIa (*P* = 0.463, Fig. 3A) and IIIb (*P* = 0.014, Fig. 3B), which indicated that the current TNM classification (7th edition) might not be suitable to the patients with RGC. Further, we deemed it might be caused by the number of lymph nodes harvested in RGC patients being much less than in GC patients (*P* < 0.001), which might affect the results of TNM classification evaluated by the number of lymph nodes status (Fig. 4).

Univariate log-rank test analysis revealed that tumor size and pLNR were significant prognostic factors for RGC. Multivariate Cox proportional hazards model analysis revealed that pLNR (HR: 2.405, *P* = 0.028; Table 3) was an independent prognostic factor to OS in RGC. Further, the correlation analysis also showed there was a significant correlation between the number of metastatic lymph nodes and the number of lymph nodes harvested in RGC patients (*r* = 0.79, *P* < 0.001). So we deemed the TNM classification evaluated by LNR status might be more suitable for RGC patients.

**Table 3:** Multivariate Cox proportional hazards regression analysis of OS in patients with RGC

Variables	HR (95% CI)	*P*-value
**Gender**		
Male	Reference	
Female	1.41(0.69–2.90)	0.301
**Age**	0.98 (0.93–1.02)	0.254
**T**		
T1	Reference	
T2	11.79 (0.89–156.00)	0.061
T3	7.72 (0.48–124.14)	0.149
T4	14.73 (0.86–253.18)	0.064
**N**		
N0	Reference	
N1	8.34 (1.59–43.80)	0.012
N2	12.38 (2.18–70.22)	0.004
N3a	8.45 (1.31–54.62)	0.025
N3b	10.35 (1.52–70.23)	0.017
**Tumor Grade**		
well	Reference	
moderately	0.49 (0.05–4.81)	0.537
poorly	0.65 (0.07–6.36)	0.71
**Chemotherapy**		
Yes	Reference	
No	0.57 (0.21–1.54)	0.264
**TNM**	3.899 (2.62–5.18)	< 0.001
**LNR**	2.40 (1.21–3.59)	0.028
**Primary malignant disease**		
No	Reference	
Yes	1.06 (0.55–2.05)	0.9

*RGC* remnant gastric cancer, *GC* gastric cancer, *T* tumor, *N* node, *M* metastasis, *LNR* lymph nodes ratio, *OS* overall survival

## Discussion

Previous studies have shown that the incidence of RGC is low, accounting for about 1% to 8% of GC [26]. In recent years, due to the popularization of screening and the continuous improvement of inspection methods, the detection rate of RGC has gradually increased, and clinicians and researchers have paid more and more attention to the disease. In this study, we found that RGC had a much worse OS than GC, although the TNM stage was similar in both groups. There was little or no difference in the OS curves of patients with IIa, IIIa, and IIIb RGC, suggesting that current TNM staging systems are unable to assess OS in patients with advanced RGC. LNR can be an independent prognostic factor in patients with RGC.

### RGC with a worse prognosis may be caused by the unsuitable classification system

The clinicopathologic characteristics and prognosis of RGC had been investigated in a few studies, which had not reached a consensus yet [10, 27, 28]. According to most reports, RGC was frequently diagnosed at an advanced stage, which caused a relatively low rate of curative resection and poor prognosis, suggesting that RGC may have distinct biological features from primary GC [29]. But some authors have compared RGC with primary GC and discovered no significant difference in survival between RGC and primary GC [30]. In most studies, the lymph node N staging of RGCs still follows the grading criteria of the UICC. However, in patients with first-time GC, postoperative lymph node drainage changes, and the lymph nodes detected by RGC cannot fully define the N stage, especially since RGC occurs after GC. The total number of postoperative lymph node dissections during re-surgery usually does not exceed 10, which is significantly less than the number of lymph nodes dissected by RGC after surgery for benign lesions. This may result in inaccurate staging. Studies have shown that the total lymph node detection rate and peritoneal lymph node metastasis rate of RGC are lower than those of primary proximal gastric cancer (PPGC), and TNM grading may not be suitable for RGC. In our study, we found the OS of RGC was much worse than GC, although the TNM stages of patients in the two groups were similar. But we deemed that this phenomenon might be caused by an unsuitable TNM classification system for RGC, for there was nearly no difference in OS curve among stage IIb, IIIa, and IIIb RGC patients, which indicated the present TNM staging system could not evaluate the OS of advanced RGC patients with lymph nodes metastasis. So our study also lent further support that it is necessary to investigate a new indicator to evaluate the lymph node status in advanced RGC.

In this study, RGC patients were divided into the RGC-B group and RGC-M group according to the nature of the initial disease. The numbers of the two groups were 57 (48.31%) and 61 (51.69%), respectively. The number of cases in the RGC-M group was slightly higher than that in the RGC-B group. This may have a great relationship with the application of proton pump inhibitors and the increase in the rate of radical resection of primary GC in recent years. By comparing the clinicopathological features of patients in the RGC-B group and the RGC-M group, it was interesting to find that the clinicopathological features of RGC-M were similar to those of RGC-B, except for the time interval from the start of the first operation. It indicated the primary disease has no effects on the characteristics of RGC. And there was no difference in tumor location between RGC-M and RGC-B also lends further support to the previous conclusion that the conception of CRS was more reasonable in the definition of RGC [6, 31]. The survival analysis of this study showed that the prognosis of patients in the RGC-B group was better than that of the patients in the RGC-M group, but the difference was not statistically significant. This may be related to the small number of samples included in this study, and there may be some bias.

### LNR was a better factor to evaluate the lymph node's status in RGC

Lymph node status is an established prognostic factor in both GC and RGC [12, 32]. But the Japanese classification of gastric carcinoma nor the TNM classification raised a special lymph node classification system for RGC till now. At present, most staging system (UICC/AJCC) stratifies the N value simply on the basis of the count of metastatic lymph nodes. But the total number of lymph nodes harvested in RGC was much less than in GC, which might affect the results of the N value based on the count of metastatic lymph nodes. Accumulated evidence showed the lymph nodes ratio (LNR) could reflect both qualitative and quantitative lymph node spread as well as the extent of disease, which might serve as an additional prognostic indicator to better evaluate the outcome of patients with advanced RGC [33–35]. In particular, we focused on the prognostic value and staging accuracy of the metastatic lymph nodes ratio (LNR) in patients with RGC undergoing curative resection, and we also found that LNR was an independent prognostic factor in RGC patients in multivariate analyses, for LNR was less influenced by the total number of lymph nodes harvested in operation. Since Okusa firstly demonstrated the impact of the “frequency of the metastases” on patients’ outcomes, there were few articles focusing on LNR in RGC to our knowledge [36]. In the present study, we reported that LNR might consider a simple, reproducible, and highly reliable prognostic factor in RGC patients. But the threshold of LNR in the TNM staging system of RGC needs a larger sample size to confirm.

There still existed some limitations to this study. First, the major limitation was its retrospective design in a single hospital. Secondly, we were unable to collect some information on the study patients, such as lifestyles, dietary preferences, and environmental features for statistical analysis, which might be important influencing factors. Further prospective studies were necessary to clarify the pathophysiology and development of RGC.

## Conclusion

In this study, we revealed important characteristics of RGC in detail. The OS of RGC patients with lymph node metastasis was much worse than GC with similar TNM stages, and LNR might consider a highly reliable indicator to evaluate the prognostic in RGC. Although the time interval from the first operation to RGC in RGC-B patients was much longer than that in RGC-M patients, the primary disease did not affect overall survival. In summary, after familiarizing the clinical-pathological characteristics and prognostic factors of RGC patients, clinicians can more accurately predict postoperative recurrence and metastasis, and better provide individualized and integrated treatment options for RGC patients.

## Data Availability

All data, materials, and operation videos used during the study are available from the corresponding author by request.
